# Different mCherry isoforms show distinct photophysical properties in FRET tandem constructs

**DOI:** 10.3389/fbioe.2025.1613582

**Published:** 2025-12-03

**Authors:** Birgit Hoffmann, Christian Melle, Christoph Biskup

**Affiliations:** Jena University Hospital, Biomolecular Photonics Group, Friedrich-Schiller-University Jena, Jena, Germany

**Keywords:** fluorescent protein, mCherry, mNeonGreen, alternative translation initiation site, isoform, FRET, FLIM, time- and spectrally-resolved fluorescence measurements

## Abstract

Fluorescent proteins are important reporter tools to investigate biological processes in the cellular environment at the molecular level. The spectrum of available fluorescent proteins has been greatly expanded by red-emitting fluorescent variants such as the commonly used mCherry. However, the presence of alternative translation initiation sites (aTIS) in mCherry allows for production of shorter protein isoforms with different properties that can bias the results of studies in which mCherry intensities are evaluated. In the present study, we used a novel approach of spectroscopic techniques, including Förster resonance energy transfer (FRET) to investigate the impact of aTIS on the photophysical properties and the functionality of mCherry in both, prokaryotic and eukaryotic expression systems. To this aim, FRET tandem constructs with different translation initiation sites, comprising mNeonGreen as donor fluorophore and mCherry as acceptor, were designed and systematically analyzed using steady-state spectroscopy, time- and spectrally-resolved fluorescence measurements, and fluorescence lifetime imaging (FLIM) based FRET measurements. The long isoforms exhibited similar photophysical properties like the full-length mCherry protein. They were also suitable FRET acceptors when coupled to mNeonGreen. The choice of translation initiation site markedly affected donor fluorescence lifetime, fluorescence intensity, and efficiency of energy transfer of the FRET constructs. Longer mCherry isoforms retained FRET acceptor functionality whereas shorter translational isoforms were non-functional, i.e., were non-fluorescent and had no effect on donor fluorescence lifetime. Our results provide insight into the implications of aTIS when using mCherry as fluorescent reporter. Overall, our study highlights the importance of considering translation initiation mechanisms in both pro- and eukaryotic systems, as they can substantially impact protein functionality and the interpretation of biological measurements.

## Introduction

Fluorescent proteins are widely used as reporters in many biological applications ranging from localization and expression studies to visualizing protein interactions in the cellular environment. Various fluorescent proteins have been developed, which differ in their photophysical properties, decay characteristics, maturation time, brightness, tendency to oligomerize, and photostability during long-time acquisition (Chudakov et al., 2010; Cranfill et al., 2016; Bajar et al., 2016). Fluorescent proteins are particularly utilized in studies that take advantage of Förster resonance energy transfer (FRET), a physical phenomenon named after the physical chemist Theodor Förster (Förster, 1946; Förster, 1948). FRET is a non-radiative process in which an excited donor fluorophore transfers part of its energy to a nearby acceptor fluorophore by dipole-dipole coupling. The efficiency of FRET depends on the overlap of donor’s emission spectrum and acceptor’s absorption spectrum, the orientation, and the distance between both fluorophores. Since the efficiency of FRET decays with the sixth power of the distance, the presence of FRET can be used as proof of molecular vicinity (Lakowicz, 2006; Biskup et al., 2007). By fusing proteins of interest with suitable donor and acceptor fluorophores, FRET enables investigating protein-protein interactions in the cellular environment with a high spatiotemporal resolution. Biosensors based on FRET are ideally suited for sensitive detection of ions, biomolecules, metabolites, and changes in membrane potential (Hochreiter et al., 2015). A commonly used FRET pair consists of the enhanced green fluorescent protein (EGFP) as donor and the red-emitting variant mCherry as acceptor fluorophore (Bajar et al., 2016; Shaner et al., 2004). However, as shown recently, the use of mCherry might be hampered by the fact that its genetic sequence contains alternative translation initiation sites (aTIS) which allow for the production of shorter protein isoforms, thereby generating substantial background fluorescence (Fages-Lartaud et al., 2022). aTIS have also been reported in fluorescent proteins, predominantly in red-emitting fluorescent proteins that were derived from mRFP1.3 that itself was obtained from its predecessor DsRed by fusing GFP-derived amino acids to the N-terminal sequence (Fages-Lartaud et al., 2022). Rather than being randomly located in the genome, aTIS play an important and evolutionary significant role in protein synthesis and are conserved across species like prokaryotes, vertebrates, insects, and yeast. aTIS allow to enhance the diversity and complexity of the proteome, are involved in a nuanced regulation of gene expression, and affect the overall translational output (Kochetov, 2008; Bazykin and Kochetov, 2011; Gao et al., 2016). Data obtained by mass spectrometry revealed that 40% of protein modifications generated by alternative splicing contain aTIS (Kroll et al., 2014). In general, the selection of translation initiation sites (TIS) is not random; rather, it is influenced by several factors like the secondary structure of the mRNA and the presence of upstream open reading frames (ORF). Moreover, nucleotides surrounding repeat regions near the start site can promote translation initiation, potentially contributing to atypical translation processes (Lee et al., 1996; Chen et al., 1994; Jin et al., 2006; Gleason et al., 2022). Specific sequence motifs and structural elements seem to enhance or inhibit the recognition of TIS so that translational efficiency is impacted. Moreover, the spatial arrangement of ribosomes can modulate the accessibility of TIS, which affects the rate of translation initiation. Computational approaches, including deep learning algorithms and artificial neural networks, have been developed to predict translation initiation sites based on genomic sequences and ribosome data (Hatzigeorgiou, 2002). Certain TIS might be preferentially utilized under distinct cellular conditions, which indicates that translational efficiency is dynamically regulated in response to environmental changes (García et al., 1995; McCutcheon et al., 1999; Petrelli et al., 2001; Elvekrog and Gonzalez, 2013; Arabfard et al., 2018; Mignone et al., 2002; Maddi et al., 2022). These findings highlight the intricate relationship between TIS and the regulatory and structural components.

In this study, we employed spectroscopic techniques to investigate the impact of translation initiation sites on the photophysical properties and the acceptor functionality of the red-emitting fluorescent protein mCherry upon expression in pro- and eukaryotes. We were especially interested in how translation initiation is affected by different expression systems. However, since aTIS can induce the production of non-fluorescent protein isoforms that cannot be detected by fluorescence techniques, an impact of aTIS on the expression of fluorescent proteins is not directly accessible. To circumvent this obstacle, we employed FRET and designed tandem constructs with different translation initiation sites, each containing a donor closely attached to an acceptor fluorophore in a 1:1 ratio. The red-emitting variant mCherry was used as acceptor fluorophore and coupled to mNeonGreen, which is a monomeric green fluorescent protein based on the tetrameric fluorescent protein LanYFP and characterized by a reduced susceptibility to environmental conditions, fast maturation half-time, an increased quantum yield of 0.8, superior brightness, and photostability as compared to EGFP (Shaner et al., 2013; Steiert et al., 2018). Due to their high expression homogeneity in protein interaction studies, both fluorophores are often used in FRET-based approaches (McCullock et al., 2020). Whenever alternative translational mCherry isoforms are expressed and exhibit altered absorption properties, the effect on FRET efficiency can be directly detected by changes in the donor emission and the donor fluorescence lifetime. We have photophysically characterized the isolated fluorescent proteins with steady-state spectroscopy and time- and spectrally-resolved fluorescence measurements. By using FLIM-FRET-based measurements, we have explored the excited-state dynamics of the fluorescent proteins and the alternative translational mCherry isoforms upon expression in eukaryotic cells. The measurements provide insight into the implications of aTIS on the observed photophysical properties, which affect the use of mCherry as a reporter and FRET acceptor in pro- and eukaryotic expression systems.

## Materials and methods

### Materials

All chemicals and reagents were purchased from Sigma Aldrich, Carl Roth, Merck, and Thermo Fisher Scientific (Germany) unless otherwise stated. The chemicals were of analytical grade or better and were used without further purification. Milli-Q-water (Millipore, United States of America) having a resistivity of 1.82 ⋅ 10^5^ Ωm at 25 °C was used for preparation of all solutions. Dye stock solutions were stored in the dark at 4 °C and working solutions were prepared immediately prior to use. Phosphate buffered saline (PBS) was prepared from NaCl (137.0 mM), KCl (2.7 mM), Na_2_HPO_4_ (6.5 mM), and KH_2_PO_4_ (1.5 mM), adjusted to a pH of 7.4 at 20 °C.

### Plasmid construction, site-directed mutagenesis, protein expression, and purification

All used fluorescent constructs were PCR amplified from a pmCherry vector (Clontech Laboratories Inc., United States of America) using distinct oligonucleotide primers each (see Supplementary Table S1 for details). Several mCherry variants possessing ORF start points on mCherry methionine positions 1, 10, 17, or 23 (i.e., mCh-M1, mCh-M10, mCh-M17, mCh-M23) were cloned between *Hind*III and *Bam*HI restriction sites of a mNeonGreen-N1 vector generating mCherry-M1-mNeonGreen, mCherry-M10-mNeonGreen, mCherry-M17-mNeonGreen, and mCherry-M23-mNeonGreen, respectively. The mNeonGreen-N1 vector was generated in the following way. The mNeonGreen sequence was cut-off from a mNeonGreen-mRuby2-FRET-10 construct, which was a gift from Michael Davidson (Addgene plasmid # 58179; http://n2t.net/addgene:58179; RRID:Addgene_58179) (Shaner et al., 2013), using *Age*I/*Not*I and cloned into a pEGFP-N1 backbone (Clontech) without the EGFP coding sequence producing a mNeonGreen vector possessing common Multiple Cloning Sites (MCS). The so produced fluorescent fusion proteins contained donor and acceptor proteins that were connected by a five amino acid linker each. Fusion constructs, in which the full-length expression of mCherry is ensured, were used as positive FRET control. To this aim, we cloned mNeonGreen between a *Hind*III restriction site of a pmCherry vector, thereby generating the mNeonGreen-mCherry (NG19C) fusion construct, in which donor and acceptor are connected by a nineteen amino acid linker. Here, the acceptor fluorophore is located at the C-terminus so that aTIS do not play a role and mCherry is fully translated. In all other constructs used in the eukaryotic expression system, mCherry is located at the N-terminus so that the influence of aTIS on the photophysical properties of the acceptor fluorophore can be studied.

For analysis of purified fluorescent proteins, mNeonGreen, mCherry, mCherry-M1-mNeonGreen, and mCherry-M10Q-mNeonGreen each labelled with a His-tag at its C-terminus were generated. To this aim, we generated a modified pET16b vector without His-tag sequence (pET16m) and used this vector to clone the distinct fluorescent constructs into the *Xho*I restriction site of the pET16m vector using oligonucleotide primers that include the 6xHis-tag (Supplementary Table S1). In this way, purification of respective fluorescent proteins and their aTIS variants was ensured. As positive FRET control, we used a fluorescent tandem construct containing mCherry and mNeonGreen (mCherry-mNeonGreen) labelled with a His-tag at its N-terminus that was cloned in pET16b using *Nde*I/*Bam*HI restriction sites. Since the His-tag is located N-terminally, only full-length proteins will be eluted; truncated variants generated via aTIS lack this tag and are not eluted. To generate a fluorescent tandem construct that contains a glutamine instead of methionine at position 10 of mCherry (mCherry-M10Q-mNeonGreen), we performed site-directed mutagenesis (QuikChange II, Agilent Technologies, Cedar Creek, TX, United States of America) as described in the instruction manual. In the generated tandem constructs, donor and acceptor were connected by a five amino acid linker each. The correct insertion of all PCR products in the respective vectors was confirmed by sequencing. For purification, the fluorescent constructs were overexpressed in the *E. coli* strain BL21. One colony of freshly transformed *E. coli* harboring the pET16m or pET16b fluorescent protein expression plasmid was used to inoculate 10 mL LB medium (supplemented with 100 μg/mL ampicillin). The culture was left overnight with shaking (235 rpm) at 37 °C. This starter culture was then filled up to 100 mL of LB medium supplemented with 100 μg/mL ampicillin and growth continued at 37 °C up to OD_600_ 0.6–0.8. Then, protein expression was induced by the addition of 0.5 mM isopropyl-1-thio-β-D-galactopyranoside (IPTG). Thereafter, growth temperature was decreased to 30 °C and cells were further cultured for 5 h. Afterwards, cells were harvested by centrifugation (10,000 × g for 15 min at 4 °C), resuspended in PBS, and centrifuged again. The cell pellet was processed immediately or stored at −80 °C. For purification of distinct fluorescent proteins, the cell pellet was resuspended using prechilled lysis buffer consisting of 100 mM sodium phosphate (pH = 7.4), 250 mM NaCl, 0.1% NP-40, supplemented with 0.01% (v/v) CHAPS, 0.1 mM phenylmethylsulfonyl fluoride (PMSF) and 1 μg/mL leupeptine. The cells were disrupted by two freezing/thawing cycles using liquid nitrogen followed by three times of sonication (Branson Sonifier II, W-250 D, G. Heinemann Ultraschall- und Labortechnik, Germany) for 30 s at 20% amplitude. The cell extract was clarified by centrifugation at 10,000 x g for 30 min at 4 °C. The solution was loaded onto Talon slurry (Talon metal affinity resin, Clontech Laboratories Inc.), which was preincubated with equilibration/wash buffer (100 mM sodium phosphate (pH = 7.4), 250 mM NaCl, 10 mM imidazole), to bind on the Talon resin using an overhead shaker for 1 h at room temperature. To elute the fluorescent protein, Talon slurry containing bound His-tagged proteins was washed twice with wash buffer followed by incubation with elution buffer (100 mM sodium phosphate (pH = 7.4), 250 mM NaCl, 300 mM imidazole) for 1 h at room temperature in an overhead shaker. Finally, eluted fractions containing fluorescent proteins were dialyzed in dialyzing buffer (100 mM sodium phosphate (pH = 7.4), 250 mM NaCl) overnight at 4 °C.

### Steady-state spectroscopy, quantum yield, and pK_a_ determination

Absorption spectra were recorded with a double-beam dual monochromator spectrophotometer (Lambda 650, PerkinElmer, Germany). All samples were measured against respective blank solutions at room temperature. Excitation and fluorescence spectra were recorded with a spectrofluorometer (QuantaMaster 30, PTI/Photomed, Germany) with excitation and emission slits set to 5 nm each. Spectra were corrected offline by subtracting the blank contributions. Ultra-micro quartz cuvettes (0.3 mm path length, Hellma Optics, Germany) were used for all measurements. Fluorescein (1 µM in 0.1 M NaOH) and coumarin 6 (1 µM in ethanol, spectroscopy grade) were used for validating instrument performance prior each measurement series. To ensure the accuracy and reliability of the fluorescence measurements, the quantum yield of the donor fluorophore was calculated relative to the fluorescence quantum yield standard fluorescein according to:

$$\Phi =\Phi_{\text{ref}}\frac{\eta^{2}}{\eta_{\text{ref}}^{2}}\frac{I}{A}\frac{A_{\text{ref}}}{I_{\text{ref}}}$$ (1)

Equation 1: Calculation of relative fluorescence quantum yield.

where Φ_ref_ is the quantum yield of the reference fluorescein dissolved in 0.1 M NaOH (Φ = 0.90), η indicates the averaged refractive indices of the solvents used for dissolving the reference (η_NaOH_ = 1.330) or the fluorescent protein (η_Tris_ = 1.335), I is the integrated fluorescence intensity, and A denotes the absorbance at the excitation wavelength (Resch-Genger, 2008; Rurack, 2008; Lide, 2005; Polyanskiy, 2022; Heiner and Osvay, 2009). The subscript “ref” refers to the reference. Reliable quantum yield determination was verified by cross calibrating the standards fluorescein and rhodamine 6G as described previously (Hoffmann et al., 2023). All samples were measured at room temperature. The Förster radius R_0_ representing the distance at which the rate of energy transfer equals the spontaneous decay rate of the donor fluorophore was calculated as follows:

$$R_{0}=0.2108\;{\left[\kappa^{2}\Phi_{D}\eta^{-4}\int_{0}^{\infty }I_{D}\left(\lambda \right)\varepsilon_{A}\left(\lambda \right)\lambda^{4}d\lambda \right]}^{\frac{1}{6}}$$ (2)

Equation 2: Calculation of Förster radius.

where κ^2^ denotes the orientation factor, Φ_D_ indicates the quantum yield of the donor fluorophore in absence of an acceptor (Φ_mNeonGreen_ = 0.8), η is the mean refractive index of the buffer (η_Tris_ = 1.335) in the respective wavelength band, I_D_ indicates the normalized fluorescence spectrum of the donor, λ is the wavelength expressed in nm, and ε_A_ is the molar extinction coefficient of the acceptor in M^−1^cm^−1^ (ε_mCherry_ = 72.000 M^−1^cm^−1^) taken from published data (Lakowicz, 2006; Shaner et al., 2004; Shaner et al., 2013; Heiner and Osvay, 2009; Valeur, 2001). To facilitate comparison with published data, the value of the averaged orientation factor was set to 2/3 under the simplified assumption that rotation of the fluorophores is unrestricted and fast as compared to the lifetime of the donor in the excited state (Valeur, 2001).

The pK_a_ values of mNeonGreen and mCherry were determined by dissolving the purified fluorescent proteins to a final concentration of 1 µM in the following pH buffered solutions in 96-well plates (Sarstedt, Germany): citrate buffer (pH < 6), phosphate buffer (pH 6–9), and glycine buffer (pH > 9). Protein solutions were excited at 450 nm (mNeonGreen) or 540 nm (mCherry) and fluorescence intensities were recorded at 520 nm or 610 nm using a plate reader (Tecan, Infinite 200Pro, United States of America). All measurements were performed in triplicate at room temperature. Fluorescence intensities were corrected offline by subtracting the blank contributions. For method validation, a solution of purified EGFP was used as control. Its pK_a_ value was determined to be 6.2, which agrees well with published data, so that reliable pK_a_ determination was ensured (Shaner et al., 2005).

### Time- and spectrally-resolved fluorescence measurements

The instrumentation used for the time- and spectrally-resolved fluorescence measurements and the data analysis routines have been described in detail elsewhere (Hoffmann et al., 2023; Biskup et al., 2004; Hoffmann et al., 2008). In brief, 1 µM fluorescent protein solutions were placed in ultra-micro cuvettes (Hellma) and excited at 488 nm (mNeonGreen) or 561 nm (mCherry) selected from a broadband supercontinuum white light laser source (SuperKExtreme, NKT Photonics, Denmark) by using an acousto-optical tunable filter (SuperK SELECT, NKT Photonics). Suitable laser clean-up filters for donor and acceptor excitation (ZET 488/10 or ZET 561/10, Chroma, AHF Analysentechnik, Germany) were inserted into a fiber-delivery system (NKT Connect, NKT Photonics) so that only a narrow wavelength band was selected whereas the rest of the continuum was effectively suppressed. The excitation light was guided via an optical fiber (FD7, NKT Photonics) to the sample placed in a cuvette holder made by our workshop. Emitted light was guided via a fiber to a spectrograph (Model 250IS, Chromex Inc., United States of America), dispersed by a holographic grating along the horizontal axis, and focused onto the entrance slit of a streak camera (Model C5680, Hamamatsu Photonics, Germany). Incident photons were converted by a photocathode (S20, Hamamatsu Photonics) into photoelectrons, which were accelerated by the streak tube, deflected by a sweep unit (M5677, Hamamatsu Photonics), and multiplied by a micro-channel plate before hitting a phosphor screen. To impose a time axis onto the spectrum, a voltage ramp was applied to the deflection electrodes. As a result, the horizontal and vertical positions of a photoelectron on the phosphor screen were determined by the wavelength and arrival time of the incident photon, respectively. A CCD camera was used to read out the optical image at the phosphor screen in 56 ms intervals. Intensities exceeding a threshold were saved in a so-called dynamic photon counting file. Streak images were analyzed using our own software written in MATLAB (R2012a-R2022a, MathWorks Inc., United States of America) and are displayed by false colors ranging from black (no counts) over green, orange to red indicating high count rates per pixel (Hoffmann et al., 2023; Biskup et al., 2004; Hoffmann et al., 2008). From the streak image data, fluorescence spectra, decay surfaces, and decay curves were extracted.

### Cell culture and transient transfection

Human embryonic kidney (Hek293) and human bone osteosarcoma epithelial (U2OS) cells were cultured in Dulbecco’s modified Eagle’s medium (DMEM, Gibco, Thermo Fisher Scientific, Germany) which was supplemented with 10% fetal calf serum (Biochrom, Germany) at 37 °C in a humidified atmosphere containing 5% CO_2_. Cells were passaged once (Hek293) or twice (U2OS) per week and grown to subconfluent density. For live-cell imaging experiments, cells were seeded on coverslips (∅ 25 mm, Menzel, Germany) coated with poly-L-lysine hydrobromide (0.1 mg/mL in sterile water) in case of Hek293 cells and placed inside cell culture dishes (∅ 60 mm, Greiner, Germany). Cells were transiently transfected using calcium phosphate precipitation based on standard protocol 24 h after seeding. Cells were used for imaging 48 h after transfection. The absence of *mycoplasma* contamination was regularly tested.

### Confocal imaging and fluorescence lifetime measurements

The TCS SP8 confocal laser scanning microscope (Leica Microsystems, Germany) equipped with a HC FLUOTAR 25x/0.95 dipping objective (Leica) was used for all imaging experiments. Confocal images were acquired before and after each lifetime measurement to verify that the fluorophores were expressed and that the specimen stayed in place during the measurement. The fluorescent proteins were excited at 488 nm (donor) or 561 nm (acceptor) selected from a supercontinuum white light laser (SuperKExtreme, NKT Photonics) operated at a repetition rate of 80 MHz by using an acousto-optical tunable filter. An acousto-optical beam splitter (AOBS) separated excitation from emission light. The fluorescence was collected within detection bands of 500–550 nm (donor) and 580–630 nm (acceptor) using the built-in hybrid detectors (Leica). To minimize overexpression artifacts, only cells with a low to medium level of expression were chosen for acquisition. The pinhole aperture was set to 1 Airy unit for all measurements. Images were analyzed with Leica LAS X (version 1.8.0.13370) software and adjusted for brightness/contrast.

For FLIM-FRET-based measurements, samples were excited at 488 nm or 561 nm using the white light laser source operated at a repetition rate of 20 MHz. The fluorescence was recorded within a spectral range of 500–550 nm or 580–630 nm and individual photon events were registered in the time-tagged time-resolved (TTTR) mode using a time-correlated single photon counting (TCSPC) module (HydraHarp 400, Picoquant, Berlin, Germany) that was directly connected to the built-in hybrid detector. For all FLIM measurements, images were reconstructed with a resolution of 512 × 512 pixels. Individual photon events were accumulated during 25 scans corresponding to a total acquisition period of 519 s. The laser intensity was adjusted such that the count rate was below 1% of the laser repetition rate.

Prior to the measurements, control experiments were performed to ensure that the setup was properly adjusted and that fluorescence lifetimes can be reliably detected. To this aim, the lifetime standards coumarin 6 (100 µM in ethanol, spectroscopy grade) and sulforhodamine 101 (1 µM in ethanol, spectroscopy grade) were filled in microscopic chambers made by our workshop. The averaged fluorescence lifetimes of coumarin 6 ( $\bar{\tau }=$ 2.52 ± 0.01 ns, n = 34) and sulforhodamine 101 ( $\bar{\tau }$ = 4.24 ± 0.01 ns, n = 46) obtained at room temperature agreed well with published values (Dutt and Raman, 2001; Brismar et al., 1995). The instrument response function of the system was estimated to be lower than 150 ps (full-width at half-maximum, FWHM) by using a non-fluorescent scattering solution (LUDOX HS-30 colloidal suspension, Sigma Aldrich). To determine the contribution of cellular autofluorescence, unstained Hek293 and U2OS cells were regularly measured. Cells that co-expressed unfused mNeonGreen and unfused mCherry were used as negative control to exclude that non-specific FRET due to protein aggregation or changes in refractive index might hamper lifetime determination.

### Data analysis

Recorded lifetime data were analyzed by using our own software written in MATLAB (R2012a-R2022a, MathWorks Inc., United States of America) and a commercial software package (SymPhoTime 64, version 2.2) from Picoquant as described previously (Hoffmann et al., 2023; Melle et al., 2024). The goodness of fit was evaluated in terms of fluctuations of residuals and reduced $\chi_{\nu }^{2}$ values. A fit was considered acceptable when the corresponding residuals were randomly distributed around zero and the $\chi_{\nu }^{2}$ value was below 1.5 (Lakowicz, 2006). Amplitude-weighted mean fluorescence lifetimes $\bar{\tau_{m}}$ were calculated as follows:

$$\bar{\tau_{m}}=\sum_{i}A_{i}\tau_{i}$$ (3)

Equation 3: Calculation of amplitude-weighted mean lifetimes.

where A_i_ is the relative amplitude of the respective decay component τ_i_. Fluorescence lifetimes are given as mean lifetimes ± standard error of the mean (SEM) with the number of measurements indicated in parentheses. Fluorescence lifetime images are shown in color-coded representation. All measurements were obtained from at least three independent experiments. By comparing the lifetime of the donor in presence of an acceptor ( $\left.\tau_{DA}\right)$ with the lifetime of the donor in absence of an acceptor ( $\tau_{D}),$ the overall efficiency of FRET was calculated as follows:

$$E=1-\frac{\tau_{DA}}{\tau_{D}}=\frac{R_{0}^{6}}{\left(R_{0}^{6}+r^{6}\right)}$$ (4)

Equation 4: Calculation of FRET efficiency.

where R_0_ is the Förster radius and r is the actual distance between the fluorophores. FRET was considered to occur when the measured sample lifetime differed by more than three standard deviations from the average of the mean lifetimes of the donor only control:

$$\tau_{m}\left(sample\right)<\bar{\tau_{m}}\left(control\right)-3\;s.d.$$ (5)

Equation 5: Calculation of FRET confidence threshold.

## Results

### Steady-state spectroscopic characterization of isolated mNeonGreen, mCherry, and FRET tandem constructs

To estimate the photophysical properties of the fluorescent proteins in absence of energy transfer, unfused mNeonGreen and mCherry were purified using standard affinity chromatography (see Materials and Methods section for details). The isolated proteins were spectroscopically characterized (Table 1). Representative steady-state absorption, excitation, and fluorescence spectra are shown in Supplementary Figure S1. Our data show that the donor fluorophore mNeonGreen exhibited an absorption maximum at 506 nm, a prominent shoulder at 476 nm, and a wide emission band with a maximum located at 520 nm. The quantum yield and the pK_a_ value were determined to be 0.7 and 5.9, respectively, which agree well with published data (Shaner et al., 2004; Steiert et al., 2018). The absorption, excitation, and emission maxima of the acceptor fluorophore mCherry were located at 587 nm, 585 nm, and 610 nm, respectively (Table 1). The maxima were identical to reported values, confirming that our chosen protocol was suitable for protein purification (Shaner et al., 2004; Merzlyak et al., 2007). A pK_a_ value of 3.7 was obtained (Shaner et al., 2004). To estimate the spectral overlap of both fluorophores and to explore the theoretical performance of the acceptor for FRET to occur, the overlap integral J(λ) and the Förster radius R_0_ were calculated yielding values of 2.4 × 10^15^ M^-1^cm^-1^nm^4^ and 57.1 Å. To determine the extent of energy transfer between the fluorophores experimentally, we generated a set of tandem constructs consisting of mNeonGreen as the donor and mCherry as the FRET acceptor in a fixed 1:1 stoichiometry. Both fluorophores were separated by a short polypeptide linker (see Materials and Methods section for details) that should allow for flexibility within the tandem constructs. In case of the mCherry-mNeonGreen construct, the absorption maxima of donor and acceptor were observed at 506 nm and 585 nm, respectively. While the emission peak of the donor moiety was still centered at 520 nm, the wavelength of maximum acceptor emission was slightly blue-shifted to 607 nm as compared to mCherry (Supplementary Figure S1).

**TABLE 1:** Steady-state spectroscopic characterization of isolated mNeonGreen, mCherry, and FRET tandem constructs.

Construct	Absorption maximum [nm]	Excitation maximum [nm]	Emission maximum [nm]
mNeonGreen	506	503	520
mCherry	587	585	610
mCherry-mNeonGreen	506	504	520
mCherry-M1-mNeonGreen	505	505	520
mCherry-M10Q-mNeonGreen	505	503	520

Previous studies revealed that methionines located at positions 1, 10, 17, and 23 of the mCherry sequence serve as aTIS in prokaryotes, which allow to produce truncated protein isoforms that might hamper the design and the interpretation of studies using mCherry as fluorescent reporter (Fages-Lartaud et al., 2022). Interestingly, mCherry seems to be functional when expressed in an N-terminal truncated variant (M10) in *E. coli* and *M. tuberculosis* whereas shorter isoforms (M17 and M23) are non-functional in prokaryotes. Furthermore, mutation of methionine to leucine of the second start codon might induce a more frequent use of the third AUG initiator codon, which itself was shown to be essential for mCherry functionality (Fages-Lartaud et al., 2022; Zelmer et al., 2012; Carroll et al., 2018; Niemeyer et al., 2023). These findings motivated us to design the FRET constructs mCherry-M1-mNeonGreen with the non-truncated mCherry (M1) and the acceptor mutant (M10Q) that exhibits an altered second translation initiator codon. The tandem construct mCherry-M1-mNeonGreen exhibited an absorption peak at 505 nm and an excitation maximum at 505 nm. Its emission spectrum closely resembled the spectral shape of mCherry-mNeonGreen (Supplementary Figure S1) with emission maxima located at 520 nm and 607 nm, which can be attributed to the donor and acceptor moiety, respectively. Since methionine plays an important role as translation initiation site in protein synthesis, we were interested how amino acid replacement would affect the translation of mCherry and how this might affect its photophysical properties (Brosnan et al., 2007). By substituting methionine with glutamine, an amino acid that was reported to preserve the structure of the fluorescent protein via hydrogen bonding and intermolecular forces, the mCherry-M10Q-mNeonGreen tandem construct was obtained (Fages-Lartaud et al., 2022). The absorption and excitation maxima of the donor moiety were located at 505 nm and 503 nm. The emission maxima were centered at 520 nm and 610 nm like in case of mNeonGreen and mCherry (Table 1).

### Time- and spectrally-resolved fluorescence measurements of isolated mNeonGreen, mCherry, and FRET tandem constructs

**FIGURE 1:**
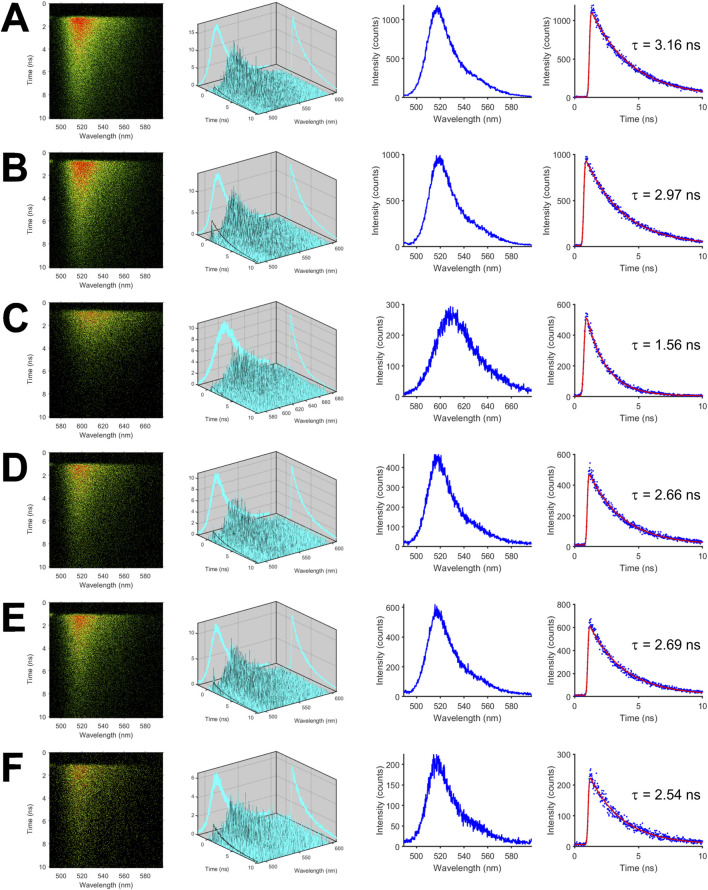
Time- and spectrally-resolved fluorescence measurements of isolated mNeonGreen, mCherry, and FRET tandem constructs. Representative streak images of mNeonGreen **(A)**, mNeonGreen in 50% glycerol/buffer mixture (v/v) **(B)**, mCherry **(C)**, the positive FRET control mCherry-mNeonGreen **(D)**, mCherry-M1-mNeonGreen **(E)**, and mCherry-M10Q-mNeonGreen **(F)** are shown in the first column. In the second to fourth columns (from left to right), decay surfaces, fluorescence spectra, and fluorescence decays are shown that were extracted from so-called dynamic photon counting (dpc) files. All measurements were performed in ultra-micro quartz cuvettes with a path length of 0.3 cm at room temperature.

To investigate the photophysical properties and the excited-state dynamics of the fluorescent proteins in more detail, we measured fluorescence in a time- and spectrally-resolved manner using our streak camera-based setup. Representative streak images of the isolated fluorescent proteins are shown in the first column of Figure 1 with the horizontal axis denoting the wavelength in nm while the vertical axis shows the time in ns. In case of mNeonGreen, the highest count rate per pixel depicted by red color was centered at the wavelength of maximum emission (Figure 1A). The second column of Figure 1 is a three-dimensional plot of the color-coded data shown in the first column. The fluorescence spectra depicted in the third column were obtained by accumulating fluorescence intensities along the time axis, i.e., the vertical axis, and plotting the resulting intensities versus the wavelength (horizontal axis). By summing up fluorescence intensities in the donor wavelength band from 500 to 550 nm, which is devoid of mCherry fluorescence (Supplementary Figure S1), and by plotting the intensities versus time, the fluorescence decay of mNeonGreen was obtained (Figure 1A, fourth column). The decay could be adequately fitted by a monoexponential model function yielding an average donor lifetime of $\bar{\tau_{m,d}}=$ (3.20 ± 0.05) ns (n = 17). To estimate how changes in the environment of the fluorophore affect lifetime, experiments in presence of glycerol (Figure 1B) were performed. A significant decrease in lifetime of mNeonGreen ( $\bar{\tau_{m,d}}=$ (2.95 ± 0.01) ns, n = 8) was observed. A representative streak image of mCherry is shown in Figure 1C, from which the fluorescence decay surface, the spectrum, and the decay have been derived. A monoexponential fit was sufficient to describe the data, which were extracted in the acceptor wavelength band from 580 to 630 nm, yielding an average acceptor lifetime of $\bar{\tau_{m,a}}=$ (1.56 ± 0.01) ns (n = 20). To test whether mCherry can be directly excited at the blue edge of its absorption spectrum, the isolated protein was excited at 488 nm, i.e., the wavelength for donor excitation. At the used laser intensities, mCherry fluorescence was not observed. As a further control, mNeonGreen was excited at 561 nm. Since mNeonGreen fluorescence was not detected (data not shown), spectral crosstalk can be excluded at our experimental conditions. The decay of the donor moiety of the positive FRET control, i.e., mCherry-mNeonGreen, was accelerated (Figure 1D). The donor lifetime was decreased to $\bar{\tau_{m,d}}=$ (2.67 ± 0.01) ns (n = 13) as compared to the donor in absence of an acceptor (Table 2). The decrease in lifetime corresponds to a FRET efficiency of 17%, which was calculated by using Equation 4. To test whether mCherry is functionally expressed, the acceptor within the tandem construct was directly excited at 561 nm. The acceptor lifetime of $\bar{\tau_{m,a}}=$ (1.61 ± 0.01) ns (n = 13) was close to mCherry, indicating that the acceptor is barely affected by the directly connected polypeptide linker and the fused donor fluorophore. Fitting the decays of the isolated mCherry-M1-mNeonGreen construct (Figure 1E) yielded a donor lifetime of $\bar{\tau_{m,d}}=$ (2.69 ± 0.01) ns (n = 15) corresponding to a FRET efficiency of 16%, which is similar to the value obtained for mCherry-mNeonGreen (Table 2). Upon excitation at 561 nm, the acceptor lifetime of mCherry-M1-mNeonGreen was determined to be $\bar{\tau_{m,a}}=$ (1.59 ± 0.01) ns (n = 8), which again is close to the lifetime of the purified mCherry. The mCherry-M10Q-mNeonGreen tandem construct (Figure 1F) was characterized by a shortened donor lifetime of $\bar{\tau_{m,d}}=$ (2.52 ± 0.02) ns (n = 19, E = 21%) as compared to mCherry-M1-mNeonGreen (Table 2). The mean acceptor lifetime of $\bar{\tau_{m,a}}=$ (1.51 ± 0.04) ns (n = 5) was in the range of mCherry, thereby indicating that the FRET acceptor within the M10Q construct is functionally expressed.

**TABLE 2:** Fluorescence lifetime data of mNeonGreen as donor fluorophore in FRET tandem constructs measured in the indicated expression systems.

Construct	Expression system	n	$\bar{\tau_{m,d}}$ [ns]	FRET efficiency [%]
mNeonGreen	*E. coli*	17	3.20 ± 0.05	-
mCherry-mNeonGreen	*E. coli*	13	2.67 ± 0.01	17
mCherry-M1-mNeonGreen	*E. coli*	15	2.69 ± 0.01	16
mCherry-M10Q-mNeonGreen	*E. coli*	19	2.52 ± 0.02	21
mNeonGreen	U2OS Hek293	19 30	3.02 ± 0.01 3.00 ± 0.01	- -
mNeonGreen + mCherry	U2OS Hek293	18 35	2.98 ± 0.01 2.99 ± 0.01	1 <1
mNeonGreen-mCherry	U2OS Hek293	11 23	2.25 ± 0.01 2.21 ± 0.02	25 26
mCherry-M1-mNeonGreen	U2OS Hek293	28 25	2.13 ± 0.02 2.16 ± 0.01	29 28
mCherry-M10-mNeonGreen	U2OS Hek293	43 33	2.34 ± 0.02 2.33 ± 0.02	23 22
mCherry-M17-mNeonGreen	U2OS Hek293	24 15	2.95 ± 0.01 2.98 ± 0.01	2 1
mCherry-M23-mNeonGreen	U2OS Hek293	18 12	2.95 ± 0.01 2.99 ± 0.02	2 <1

The number of measurements or cells used for lifetime determination is shown. Fluorescence lifetimes are given as mean lifetimes ± SEM. FRET efficiencies are calculated from donor mean lifetimes in presence and in absence of an acceptor according to Equation 4.

### FLIM-FRET-based control measurements in eukaryotic expression systems

**FIGURE 2:**
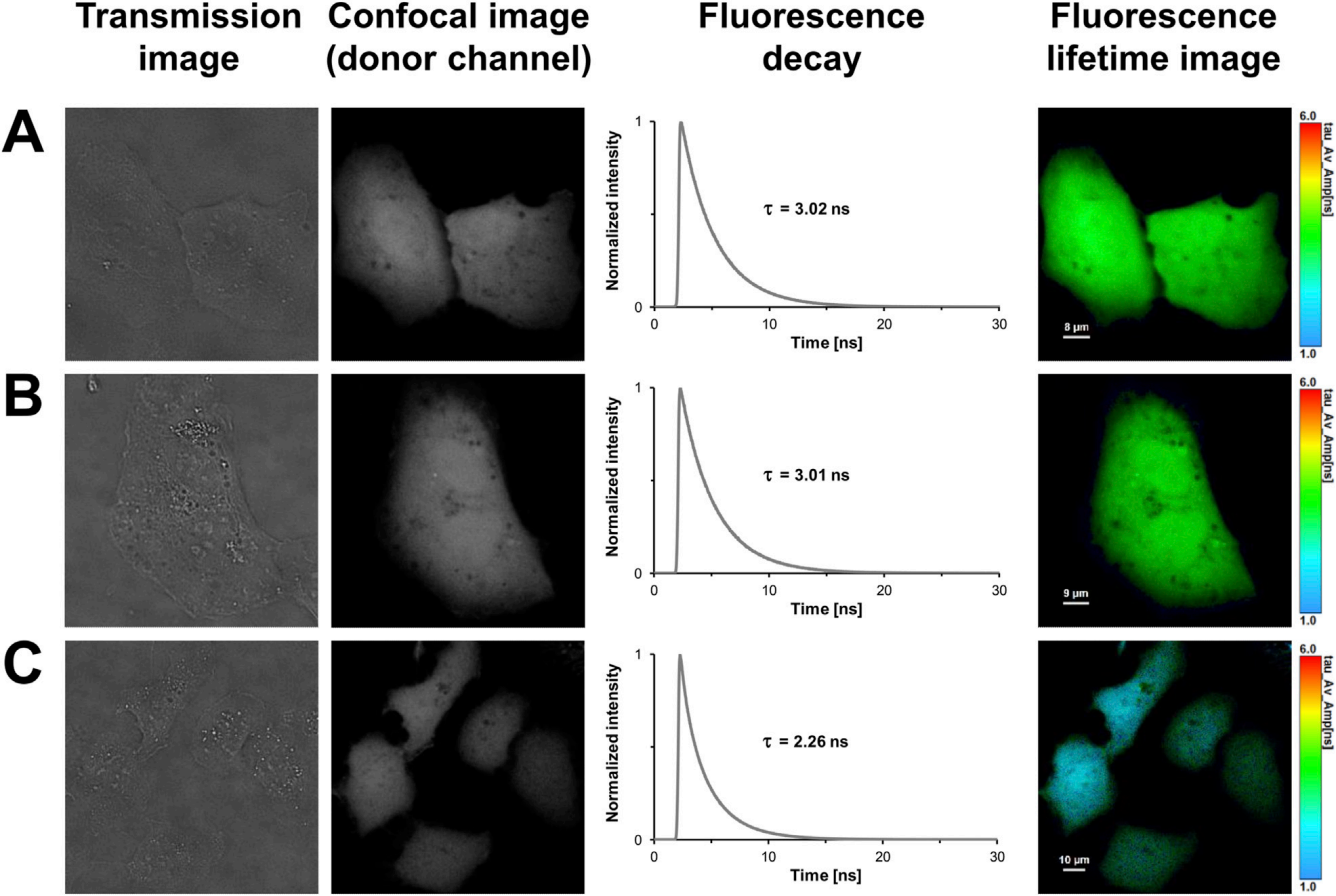
FLIM-FRET-based control measurements of U2OS cells expressing the donor mNeonGreen in absence or presence of the acceptor mCherry, and the positive FRET control. In the first column, representative transmission images of U2OS cells expressing mNeonGreen **(A)**, unfused mNeonGreen and unfused mCherry **(B)**, and the positive FRET control mNeonGreen-mCherry **(C)** are shown. In the second column, grayscale confocal images are displayed that were obtained in the donor channel by exciting at 488 nm and recording fluorescence within a detection band of 500–550 nm. The third column shows the fluorescence decay curves of the cells. In the last column, fluorescence lifetime images are shown, in which lifetimes are encoded by color as specified by the color scale. Lengths of scale bars are indicated.

Until now, aTIS generating mCherry isoforms have been mainly investigated in prokaryotic expression systems. This encouraged us to further investigate tandem constructs differing in their translation initiator codons in eukaryotic expression systems. We utilized our FLIM setup for measurements in living cells. The advantage of this setup is that, unlike with the streak camera setup, lifetimes can be determined with a high spatial resolution. This allows to detect heterogeneities of lifetimes that may result from aggregation or accumulation of the fluorescent proteins in compartments of a cell. However, the high spatial resolution comes at the expense of the spectral resolution. In the confocal laser scanning microscope donor and acceptor fluorescence are sampled in only two channels. To validate our FLIM setup and to characterize the fluorescent proteins in absence of energy transfer, a series of control experiments were performed. The first and second columns of Figure 2 show representative transmission and confocal images of U2OS cells transiently transfected with the respective fluorescent proteins. As can be seen in the images, all fluorescent proteins were homogeneously distributed throughout the cytoplasm. An aggregation or an accumulation in the endoplasmic reticulum due to overexpressed proteins was not observed. The uniform coloring of the lifetime image illustrates that the spatial variation in lifetime was low within the mNeonGreen expressing cells (Figure 2A). Like in case of the isolated protein, the fluorescence decay could be best fitted with a monoexponential model function yielding an average donor mean lifetime of $\bar{\tau_{m,d}}=($ 3.02 ± 0.01) ns (n = 19), which is concordant with published data (McCullock et al., 2020). To explore cell-line specific effects on the lifetime, mNeonGreen was transfected into Hek293 cells (Supplementary Figure S2A). The average fluorescence lifetime ( $\bar{\tau_{m,d}}=$ (3.00 ± 0.01) ns, n = 30) coincided with the value measured in U2OS cells so that an effect of cellular environment on lifetime appears to be unlikely in studied cell lines. To estimate whether random diffusion, stickiness, and weak interactions between the fluorophores might bias energy transfer estimation, cells co-expressing unfused mNeonGreen and unfused mCherry were used as negative control (Figure 2B; Supplementary Figure S2B). Lifetimes close to the unfused donor fluorophore were obtained in U2OS $\left(\bar{\tau_{m,d}}=\right.$ (2.98 ± 0.01) ns, n = 18) and in Hek293 cells $\left(\bar{\tau_{m,d}}=\right.$ (2.99 ± 0.01) ns, n = 35), indicating that unspecific FRET due to molecular crowding or unspecific associations of the fluorescent tags is negligible at the used expression levels. While a monoexponential function was sufficient to fit the recorded fluorescence decays of unfused mNeonGreen co-expressed with unfused mCherry, the decays of mNeonGreen-mCherry expressing cells had to be fitted biexponentially. A markedly reduced donor lifetime of $\bar{\tau_{m,d}}=$ (2.25 ± 0.01) ns (n = 11, E = 25%) was measured as illustrated by the blueish coloring of the lifetime image (Figure 2C). In Hek293 cells, a comparable mean lifetime of $\bar{\tau_{m,d}}=$ (2.21 ± 0.02) ns (n = 23) and a FRET efficiency (E) of 26% were obtained (Supplementary Figure S2C; Table 2).

### FLIM-FRET-based measurements of FRET tandem constructs in eukaryotic expression systems

**FIGURE 3:**
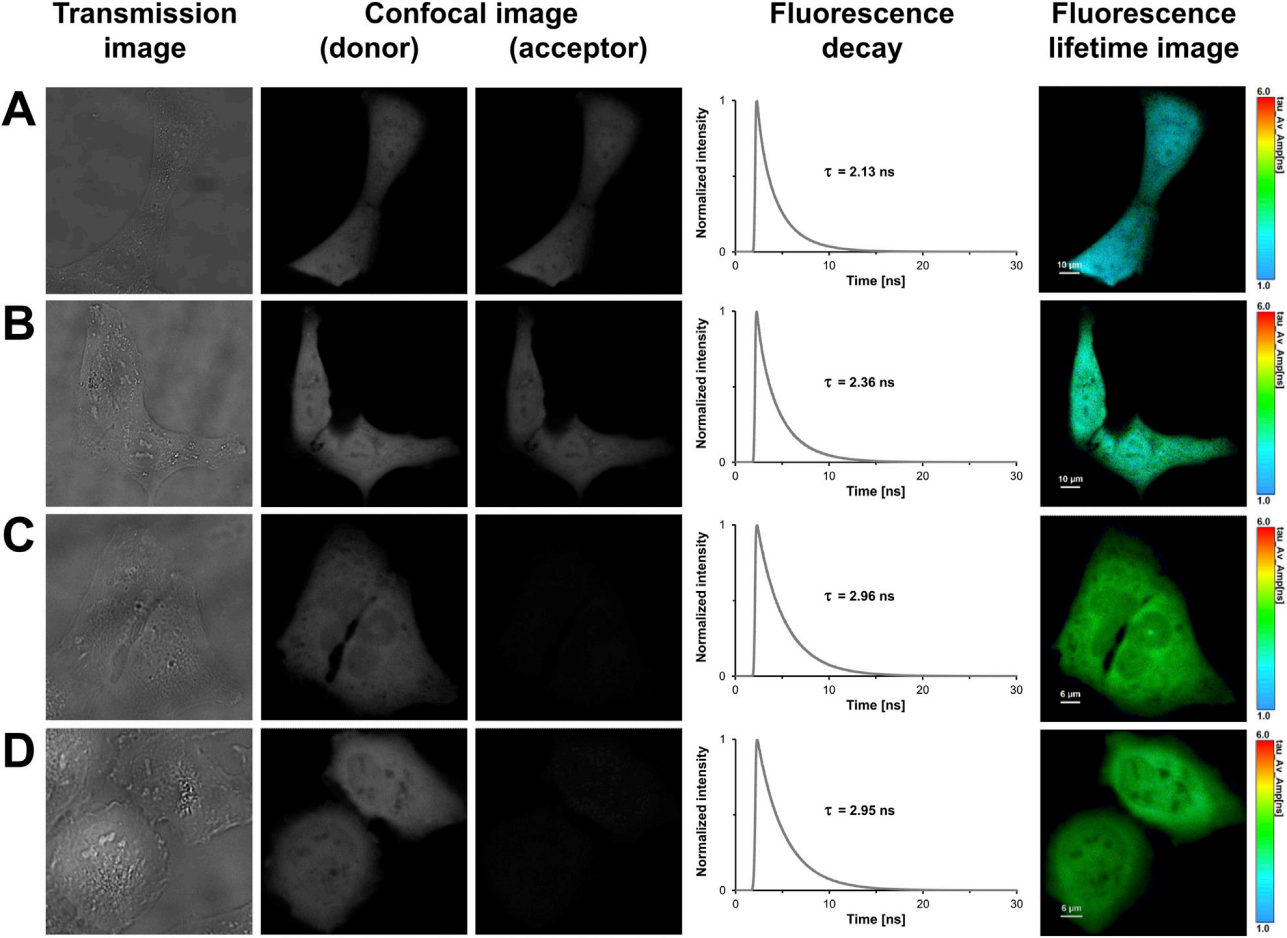
FLIM-FRET-based measurements of U2OS cells expressing FRET tandem constructs with different translation initiation sites. In the first column, representative transmission images of U2OS cells expressing mCherry-M1-mNeonGreen **(A)**, mCherry-M10-mNeonGreen **(B)**, mCherry-M17-mNeonGreen **(C)**, and mCherry-M23-mNeonGreen **(D)** are shown. The second and third columns show grayscale confocal images that were obtained in the donor and acceptor channel by recording fluorescence intensities within a detection band of 500–550 nm and 580–630 nm, respectively. The fourth column shows the fluorescence decay curves of the cells. In the last column, fluorescence lifetime images are shown, in which lifetimes are encoded by color as specified by the color scale. Lengths of scale bars are indicated.

In light of previous work and our findings in prokaryotes, we designed tandem constructs differing in their translation initiation sites, i.e., M1, M10, M17 or M23, to investigate their effect on the photophysical properties and the acceptor functionality of mCherry in eukaryotes (Fages-Lartaud et al., 2022). The confocal images shown in Figure 3 illustrate that all tandem constructs were homogeneously distributed within the U2OS cells, and that molecular crowding did not affect the properties of the fluorophores. The spatial variation in lifetime was low as shown by the uniform lifetime distribution observed in cells expressing mCherry-M1-mNeonGreen (Figure 3A). The measured fluorescence decays had to be fitted by using a triexponential function yielding an averaged donor mean lifetime of $\bar{\tau_{m,d}}=$ (2.13 ± 0.02) ns (n = 28, Table 2). To estimate the fractions of donor fluorophores engaged in energy transfer, a detailed lifetime analysis was performed revealing decay components of 3.0 ns, 2.0 ns, and 0.6 ns with fractional amplitudes of 37%, 46%, and 16%, respectively (Table 3). The slow decay component (3.0 ns) was almost identical to the mean lifetime of unfused mNeonGreen in absence of an acceptor and can be attributed to a population of unquenched donor molecules not involved in energy transfer that are characterized by FRET efficiencies close to zero. In contrast, the predominant decay component of 2.0 ns was in the range of the positive FRET control and can be attributed to mNeonGreen molecules exhibiting efficient energy transfer. The accelerated decay component of 0.6 ns might arise from a population of donor molecules characterized by high FRET. To exclude that the fast decay component might originate from autofluorescence, control measurements with unstained cells were performed. The measurements revealed that signals with short lifetimes were absent at the used experimental conditions (data not shown). To test for generalizability of our results, the mCherry-M1-mNeonGreen construct was transiently transfected into Hek293 cells (Supplementary Figure S3A). Similar values of lifetime ( $\bar{\tau_{m,d}}=$ (2.16 ± 0.01) ns, n = 25, Table 2) and decay components (Table 3) were obtained so that cell-type specific effects on the translation initiation machinery seem to be unlikely at the used expression levels. The average acceptor lifetime of $\bar{\tau_{m,a}}=$ (1.43 ± 0.01) ns (n = 18) was close to the value obtained upon expression of unfused mCherry in U2OS ( $\bar{\tau_{m,a}}=$ (1.46 ± 0.01) ns, n = 18) and in Hek293 cells ( $\bar{\tau_{m,a}}=$ (1.43 ± 0.01) ns, n = 41). To investigate whether the second AUG initiator codon in the mCherry sequence allows to produce a shorter, but still functional protein isoform in eukaryotes, the N-terminally truncated mCherry-M10-mNeonGreen construct was transiently transfected into U2OS cells (Figure 3B). Its donor lifetime was determined to be $\bar{\tau_{m,d}}=$ (2.34 ± 0.02) ns (n = 43, Table 2). The extracted decay components of 3.1 ns (48%), 2.0 ns (39%), and 0.6 ns (13%) closely resembled the values obtained for the mCherry-M1-mNeonGreen construct (Table 3). The results obtained in U2OS cells were consistent with the measurements in Hek293 cells (Supplementary Figure S3B) regarding the average mean donor lifetime ( $\bar{\tau_{m,d}}=$ (2.33 ± 0.02) ns, n = 33, Table 2) and decay components (Table 3). An acceptor lifetime of $\bar{\tau_{m,a}}=$ (1.42 ± 0.01) ns (n = 24) close to the control value of unfused mCherry was measured.

**TABLE 3:** Donor fluorescence lifetime data of the indicated constructs measured in U2OS and Hek293 cells.

Construct	Expression system	Fit	A_1_ [%]	τ_1_ [ns]	A_2_ [%]	τ_2_ [ns]	A_3_ [%]	τ_3_ [ns]	$\chi_{\nu }^{2}$
mCherry-M1-mNeonGreen	U2OS Hek293	tri-exp. tri-exp.	37.2 34.5	3.0 3.1	46.4 50.1	2.0 2.0	16.4 15.4	0.6 0.7	1.1 1.0
mCherry-M10-mNeonGreen	U2OS Hek293	tri-exp. tri-exp.	47.9 43.8	3.1 3.1	39.4 43.0	2.0 2.0	12.7 13.2	0.6 0.7	1.0 1.0
mCherry-M17-mNeonGreen	U2OS Hek293	bi-exp. bi-exp.	78.2 87.3	3.2 3.1	21.8 12.7	2.1 2.0			1.1 1.0
	U2OS Hek293	tri-exp. tri-exp.	78.4 80.7	3.1 3.1	20.0 18.3	2.3 2.3	1.6 1.0	0.6 0.5	1.0 1.0
mCherry-M23-mNeonGreen	U2OS Hek293	bi-exp. bi-exp.	86.0 84.7	3.1 3.1	14.0 15.3	2.0 2.0			1.1 1.0
	U2OS Hek293	tri-exp. tri-exp.	80.5 79.9	3.1 3.1	18.1 18.8	2.3 2.3	1.4 1.3	0.5 0.5	1.1 1.0

Mean values of fractional amplitudes, lifetimes of the respective decay components, and reduced $\chi_{\nu }^{2}$ used for estimation of the goodness of fit are shown.

In contrast to the longer isoforms, i.e., M1 and M10, the acceptor fluorescence was markedly diminished in U2OS cells expressing the shorter translational isoforms, where translation was restricted to start at M17 or M23 (Figures 3C,D). For the mCherry-M17-mNeonGreen construct, an averaged donor mean lifetime of $\bar{\tau_{m,d}}=$ (2.95 ± 0.01) ns (n = 24) was calculated (Table 2). The slow and fast decay components of 3.2 ns (78%) and 2.1 ns (22%) were in the range of the lifetime values of unfused mNeonGreen and the positive FRET control (Table 3). Similar results were obtained upon expression in Hek293 cells ( $\bar{\tau_{m,d}}=$ (2.98 ± 0.01) ns, n = 15) as shown in Tables 2, 3; Supplementary Figure S3C. The measured acceptor lifetime of $\bar{\tau_{m,a}}=$ (1.74 ± 0.05) ns (n = 7) differed significantly from mCherry, suggesting that the acceptor fluorophore was altered. Upon mCherry-M23-mNeonGreen expression (Figure 3D; Supplementary Figure S3D), an average fluorescence lifetime close to the donor only sample was measured in U2OS ( $\bar{\tau_{m,d}}=$ (2.95 ± 0.01) ns, n = 18) and in Hek293 cells ( $\bar{\tau_{m,d}}=$ (2.99 ± 0.02) ns, n = 12) with the slow decay component being predominant (Table 3). The prolonged acceptor lifetime ( $\bar{\tau_{m,a}}=$ (1.76 ± 0.04) ns, n = 9) was similar to the value measured for the mCherry-M17-mNeonGreen construct. Taken together, our results indicate that the truncated isoforms M17 and M23 display different photophysical properties, which is concordant with findings obtained in prokaryotes (Fages-Lartaud et al., 2022). To facilitate comparison with the results of the long translational isoforms, decays of cells expressing the M17 or M23 constructs were as well fitted triexponentially. The quality of the fit was not significantly improved by a higher number of fit parameters as revealed by only slightly lowered mean $\chi_{\nu }^{2}$ values for the former (1.04 vs. 1.05) and for the latter (1.07 vs. 1.08) construct (Table 3). In case of mCherry-M17-mNeonGreen, the averaged donor mean lifetimes obtained in U2OS ( $\bar{\tau_{m,d}}=$ (2.94 ± 0.01) ns, n = 24) and in Hek293 cells ( $\bar{\tau_{m,d}}=$ (2.97 ± 0.01) ns, n = 15) were quite similar and differed by approximately 1% from the lifetimes estimated by a biexponential fit. The slow decay component of 3.1 ns was predominant in both cell lines (Table 3). U2OS cells transiently transfected with mCherry-M23-mNeonGreen displayed an averaged donor mean lifetime of $\bar{\tau_{m,d}}=$ (2.93 ± 0.01) ns (n = 18) that agreed well with the lifetime of $\bar{\tau_{m,d}}=$ (2.95 ± 0.02) ns (n = 11) measured in Hek293 cells. Like in case of the M17 construct, the slow time constant (3.1 ns) was the major decay component. Overall, the short isoforms exhibited almost identical decay components and fractional amplitudes upon expression in both cell lines (Table 3). Interestingly, the values of the slow and the fastest decay component were almost identical in all studied tandem constructs.

## Discussion

Fluorescent proteins are widely used to label proteins of interest by taking advantage of the toolkit of the molecular biologist. Using these tools, unlike chemical methods, the labelling efficiency is typically expected to approach 100%. The only limitation that may give rise to a fraction of unlabeled proteins is the slow maturation time of some fluorescent proteins. Another aspect that could potentially compromise the efficiency of labeling a protein with a specific tag is the presence of aTIS that may allow to produce fluorescent proteins with different properties. Nevertheless, quantifying the expression of proteins with aTIS presents a challenge, especially when aTIS affect the fluorescence properties of the fluorophore. In this study, we utilized Förster resonance energy transfer (FRET) to investigate the effect of aTIS on the photophysical properties and the FRET acceptor functionality of mCherry in both prokaryotic and eukaryotic expression systems. To this aim, FRET tandem constructs with different translation initiation sites were generated. The fluorescent protein mCherry was used as FRET acceptor and coupled to the donor fluorophore mNeonGreen. All fluorescent proteins and constructs were systematically analyzed using steady-state spectroscopy, time- and spectrally-resolved fluorescence measurements, and FRET measurements. The extent of energy transfer was estimated by using fluorescence lifetime imaging (FLIM), which is an extremely robust and reliable method to assess FRET since the lifetime is insensitive to variations of fluorophore concentration, expression level, or absorption along the optical light path (Lakowicz, 2006; Valeur, 2001). We first studied the photophysical properties of the isolated proteins in absence of energy transfer. The absorption, excitation, and emission maxima as well as the pK_a_ values of mNeonGreen and mCherry were similar to published values. The fluorescence decay of the isolated donor fluorophore could be fitted using a monoexponential model function and its lifetime agreed well with recently published data (Clancy et al., 2023). Experiments in presence of glycerol revealed that the lifetime of mNeonGreen is sensitive to its environment, i.e., changes in refractive index and viscosity, thereby confirming previous results (Lakowicz, 2006; Valeur, 2001; Clancy et al., 2023; Suhling et al., 2001). The fluorescence decay of mCherry could be adequately described by a monoexponential function and its fluorescence lifetime was consistent with published value (Merzlyak et al., 2007; Clancy et al., 2023; Drobizhev et al., 2009; Seefeldt et al., 2008). Both fluorophores exhibited sufficient spectral overlap between donor emission and acceptor absorption for FRET to occur. The calculated Förster radius of 57 Å was slightly larger than the value of the FRET pair EGFP and mCherry (51 Å), which can be mainly attributed to the higher quantum yield of mNeonGreen as compared to EGFP (Albertazzi et al., 2009). In this context, Förster radii should be regarded as approximates of a distance distribution rather than exact distances. Moreover, fluorescent proteins are large and tend to rotate slowly during the donor’s excited state, so that the common assumption of the mean κ^2^ value of 2/3 is only a rough estimate for all possible dipole orientations (Vogel et al., 2014). In case of the positive FRET control mCherry-mNeonGreen, the spectral shape and the shortened donor lifetime demonstrated that efficient energy transfer takes place between both fluorophores, which were separated by a short amino acid linker in a fixed 1:1 ratio. The polypeptide linker was flexible enough to permit segmental rotation without restricting possible dipole orientations of the fluorophores. The spectral characteristics of the mCherry-M1-mNeonGreen and the mCherry-M10Q-mNeonGreen constructs confirmed that functional mCherry variants are produced from M1 or M10Q in prokaryotes and that the photophysical properties of the mCherry chromophore were barely affected by the M10Q substitution which is located outside of the β-barrel structure (Fages-Lartaud et al., 2022). Since methionine is absent from the sequence of the M10Q mutant, the first initiator codon (M1) seems to be more frequently used, which is in line with the measured data. Moreover, a ribosome binding site-like sequence close to the initiator codon in position 10 might further contribute to translation initiation as observed in *M. tuberculosis* (Carroll et al., 2014). Overall, the calculated energy transfer efficiencies of the FRET constructs were in the range of a tandem construct consisting of mNeonGreen and mCherry separated by a relatively short amino acid linker (McCullock et al., 2020). The acceptor lifetimes of the tandem constructs closely resembled the value of unfused mCherry. Altogether, our findings show that mCherry and its isoforms are suitable FRET acceptors when coupled to mNeonGreen, which is consistent with previously published data (McCullock et al., 2020; Mastop et al., 2017).

Interestingly, our experiments show that alternative translational mCherry isoforms are as well expressed in eukaryotes. A cell-specific effect on translation initiation was absent as verified upon expression in different cell lines. The decay of the donor fluorophore mNeonGreen could be well approximated by a monoexponential model function in both cell lines and its fluorescence lifetime agreed well with published data (Shaner et al., 2013). The donor lifetime was slightly shorter as compared to the isolated protein, which might be due to the different environment and the fact that efficiency of maturation and/or folding might vary between mammalian and bacterial cells (Lakowicz, 2006; Shaner et al., 2005). Measurements of our negative FRET control, i.e., cells co-expressing unfused mNeonGreen and unfused mCherry, demonstrated that unspecific FRET due to molecular crowding, random diffusion or weak interactions of the fluorescent proteins can be neglected at our expression levels. Due to efficient energy transfer between the closely attached fluorophores, the donor lifetime of the positive FRET control was significantly reduced as compared to the unfused donor sample. The calculated FRET efficiency differed slightly from the isolated protein, which might result from an impact of the refractive index, viscosity, and local environment on the lifetime (Lakowicz, 2006). The shortened donor lifetimes of the mCherry-M1-mNeonGreen and mCherry-M10-mNeonGreen constructs confirmed that both mCherry isoforms are suitable and functional FRET acceptors in eukaryotes. As compared to the former construct, an enlarged fraction of donor fluorophores not engaged in FRET was present in case of the mCherry-M10-mNeonGreen construct as seen by a change in the amplitude A1 from 37% to 48% in U2OS and from 35% to 44% in Hek293 cells (Table 3). The measured acceptor lifetimes were close to the lifetime of unfused mCherry, demonstrating that the fluorophore is functional even when an alternative translational mCherry isoform (M10) is expressed. Contrarily, the shorter translational isoforms M17 and M23 were essentially non-fluorescent. The donor lifetimes of both constructs were in the range of unfused mNeonGreen, indicating that none of the truncated mCherry isoforms could serve as acceptor. Prolonged acceptor lifetimes that differed significantly from unfused mCherry further confirmed that the fluorophore was affected by the truncation, which supports initial findings obtained in prokaryotic expression systems (Fages-Lartaud et al., 2022).

Since hampered functionality prevents mCherry fluorophores from acting as suitable FRET acceptor, closely attached mNeonGreen molecules are not able to transfer part of its energy via FRET (Vogel et al., 2012). Consequently, the fraction of unquenched donor molecules with lifetimes similar to the unfused donor increases. The question arises which factors might contribute to the altered functionality of truncated mCherry isoforms as observed in pro- as well as in eukaryotic expression systems. One reason might be the presence of incorrectly folded fluorescent proteins. Since many proteins fold co-translationally, short protein isoforms might be severely impaired in their folding. Due to missing structural elements, isoforms might fold improperly or less efficiently resulting in a reduced protein yield (Fages-Lartaud et al., 2022; Zutz et al., 2021). Especially upon heterologous expression, the occurrence of misfolded proteins is often related to overexpression. Since low to medium expressing cells were considered for data analysis only, the impact of expression level on acceptor functionality is negligible in the data shown in this study. However, we cannot exclude that the presence of incorrectly folded proteins, which might be incapable of acting as an acceptor, could have influenced our FRET estimation, particularly in the prokaryotic expression system. Besides, aTIS might affect the stability of the protein itself, thereby impacting fluorescence properties (Zutz et al., 2021; Wong and Truong, 2010). Protein modeling studies suggest that amino acids between the second and third initiator codon play an important role in stabilizing the mCherry protein. In addition, an interaction between M10 and a tyrosine residue (Y43) seems to further contribute to stabilization of the acceptor protein (Fages-Lartaud et al., 2022). Accordingly, the longer isoforms might be better stabilized than the shorter translational isoforms, which is in line with our findings that the M17 and M23 isoforms were essentially non-functional and exhibited altered photophysical properties. Apart from stabilization, the properties of the acceptor protein might be affected by photophysical reactions such as switching between a fluorescent and a dark state. Hereby, the fraction of fluorescent proteins able to act as reasonable FRET acceptors decreases. Considerable dark fractions of more than 50% have been reported for red-emitting fluorescent proteins that might explain their low quantum yields as compared to green-emitting variants such as EGFP or mNeonGreen. It has been suggested that a fraction of 30% remains permanently in a long-lived dark state (Heesink et al., 2022). In case of red-emitting proteins, the dark states seem to be photoreactive, suggesting a nuanced interplay between photoconversion, irreversible photobleaching, and mode of illumination. Surprisingly, mCherry photobleaches to a much lower extent from its dark states when using pulsed excitation as compared to a continuous illumination mode at the same intensity level (Dean et al., 2011). Since a pulsed white light laser with the lowest intensity possible was used for our donor and acceptor lifetime measurements, an impact of laser intensity on light-induced processes such as irreversible photobleaching and transitions into dark states was minimized. Interestingly, red-emitting proteins seem to display a pH-sensitive conversion from dark into bright states. The accompanied photophysical reactions occur on a timescale of µs to ms that is significantly longer than the lifetime of the studied proteins (Dean et al., 2011; Hendrix et al., 2008). Since all measurements were performed in buffered solutions, an effect of pH on functionality and photophysical properties of mCherry along with its translational isoforms can be excluded. Furthermore, substances that can induce chemical caging such as β-mercaptoethanol were not used in our experiments, which might explain the loss of functionality due to conversion into a blue-fluorescent intermediate (Cloin et al., 2017). Another explanation for the altered functionality of the short truncated mCherry isoforms might be an absent, slow or even hindered rotation of the fluorophores within the tandem constructs, so that energy transfer might be impeded due to large variations in fluorophore distances and unfavorable dipole orientations (Vogel et al., 2012). However, spectroscopic measurements with the long functional isoforms confirmed that the linker allows for flexibility, so that an impact of sterical hindrance on functionality seems to be negligible. It can be further assumed that a high degree of flexibility within the tandem construct permits conformational dynamics (Hendrix et al., 2008). Also, fast internal conversion might offer a competing de-excitation path and might explain the loss of fluorescence observed upon expression of the shorter isoforms. A detailed lifetime analysis revealed that fractions of donor fluorophores engaged and not engaged in energy transfer are present in the FRET constructs. By calculating the FRET efficiencies of the respective time constants (Table 3) and taking the averaged Förster radius that was spectroscopically determined, the distances between donor and acceptor within the mCherry-M1-mNeonGreen construct can be roughly estimated to be 64 Å and 45 Å at low and high FRET conditions, respectively. Surprisingly, the short time constant representing high FRET condition remained almost constant for all studied constructs (Table 3), which might indicate the smallest intramolecular distance between the fluorophores that is in the range of the minimum separation value (∼30 Å) of two fluorescent proteins (George Abraham et al., 2015). Thus, our results suggest that the donor-to-acceptor distances vary due to the inherent flexibility of the polypeptide linker, which is in line with measurements of tandem constructs consisting of cyan- and yellow-emitting proteins (Vogel et al., 2014). It is conceivable that the distribution of orientation factors might be constrained within the tandem constructs due to structural characteristics of the linker and the rigid barrels of the fluorophores themselves. Molecular dynamics simulations suggest that orientation factor and FRET distance are correlated, in contrast to the expectation that both variables are independent as anticipated in numerous FRET studies (Vogel et al., 2012; VanBeek et al., 2007). Another explanation for the impaired FRET acceptor functionality might be that maturation time of the translational isoforms differs from mCherry. Since all lifetime measurements were performed 48 h post transfection, most fluorophores should be successfully matured, so that an effect of maturation seems to be unlikely.

In general, since targeting of proteins is often mediated by N-terminal localization signals, the cellular distribution of N-terminal truncated protein isoforms might be hampered as compared to the full-length proteins (Danpure, 1995). Thus, the interpretation of localization, protein dynamics, and protein interactions might be biased by the presence of aTIS when fluorescent proteins are employed as labels. Gene expression studies might be impeded by aTIS because expression levels of the target gene are overestimated, so that conclusions from these studies might be misleading, when using fluorescence as reporter. To address the issues related to aTIS and to increase the reliability of red-emitting fluorescent proteins as reporters, engineered mCherry variants were developed that should exhibit minimized background signals. The variant obtained by substituting methionine at position 10 by glutamine resulted in a functional and properly folded fluorescent protein that seems to be useful in C-terminal fusions (Fages-Lartaud et al., 2022). Another solution might be to re-engineer or de-optimize mCherry to decrease the expression of alternative translational isoforms. A mCherry variant exhibiting properties of its predecessor mRFP1.1 was reported to be more reliable in expression and imaging studies, thereby increasing the accuracy of the results. Unfortunately, its performance was low in C-terminal fusions and some of the de-optimized variants exhibited increased background fluorescence (Fages-Lartaud et al., 2022). Based on our experiments we propose that care must be taken when using mCherry and DsRed derived fluorescent proteins due to the production of shorter isoforms that interfere with the protein of interest in pro- and in eukaryotic expression systems. Our results demonstrate that spectroscopic techniques including FLIM-FRET-based measurements are suitable and promising methods for detecting and identifying alternative translational isoforms. While the impact of aTIS has been reported in red-emitting proteins, their effects in other visible fluorescent proteins are less documented, which emphasizes the need for careful consideration before using any fluorescent tag as reporter. Further studies are necessary to quantify how prevalent aTIS are across different fluorescent proteins. This might help to understand the translation machinery and might assist in developing more accurate fluorescent proteins for research purposes. Formalized procedures including translation rate prediction might be beneficial in improving and standardizing the performance of fluorescent proteins (Mertens and den Blaauwen, 2022; Zhang et al., 2022; Trulley et al., 2019; Newman et al., 2011). Overall, our study highlights to be aware of translation initiation mechanism in both pro- and eukaryotes since aTIS can significantly impact protein functionality and the interpretation of biological measurements.

## Data Availability

The original contributions presented in the study are included in the article/Supplementary Material, further inquiries can be directed to the corresponding author.

## Abbreviations

FRET, Förster resonance energy transfer; FLIM, fluorescence lifetime imaging; EGFP, enhanced green fluorescent protein; aTIS, alternative translation initiation site; TIS, translation initiation site; PBS, phosphate buffered saline.
