# Supplementary material for: Different mCherry isoforms show distinct photophysical properties in FRET tandem constructs
Source: Front Bioeng Biotechnol. 2025 Dec 3;13:1613582. doi: 10.3389/fbioe.2025.1613582 (PMC12708940; doi:10.3389/fbioe.2025.1613582)
Supplement: Supplementary file 1 [file DataSheet1.pdf]

Supplementary Material

1 Supplementary Tables and Figures

1.1 Supplementary Tables

Supplementary Table 1. Oligonucleotide primers for PCR (both amplification and mutation).

	Forward primer (5´- 3´)	Reverse primer (5´- 3´)
mCherry-M1 (in mNeonGreen-N)	agctaagcttatggtgagcaagggcgagg	agctggatccagcttgtacagctcgtccatgcc
mCherry-M10 (in mNeonGreen-N)	agctaagcttatggccatcatcaaggagttc	agctggatccagcttgtacagctcgtccatgcc
mCherry-M17 (in mNeonGreen-N)	agctaagcttatgcgcttcaaggtgcacatgg	agctggatccagcttgtacagctcgtccatgcc
mCherry-M23 (in mNeonGreen-N)	agctaagcttatggagggctccgtgaacggc	agctggatccagcttgtacagctcgtccatgcc
Positive control NG19C (in mCherry-N1)	actgaagccttatggtgagcaagggcgaggag	cagtaagcttcttgtacagctcgtccatgcc
mCherry-M1-NG (in pET16m)	acgtctcgagtgagcaagggcgaggagg	acgtctcgagttagtgatgatgatgatgatgc ttgtacagctcgtccatgcccatcacatcgg
NeonGreen (in pET16m)	acgtggatccgtgagcaagggcgaggagg	acgtggatccttagtgatgatgatgatgatgc ttgtacagctcgtccatgcccatcacatcgg
mCherry-M10Q-NeonGreen (in pET16m)	ggcgaggaggataaccagggcatcatcaaggag	ctccttgatgatgccctggttatcctcctcgcc
mCherry (in pET16m)	acgtggatccgtgagcaagggcgaggagg	acgtggatccttagtgatgatgatgatgatgctt gtacagctcgtccatgccgccggtggagtggcg
Positive control mCherry-NeonGreen (in pET16b)	acgtcatatggtgagcaagggcgaggag	acgtggatccttagtgatgatgatgatgatgctt gtacagctcgtccatgccgccggtggagtggcg

1.2 Supplementary Figures

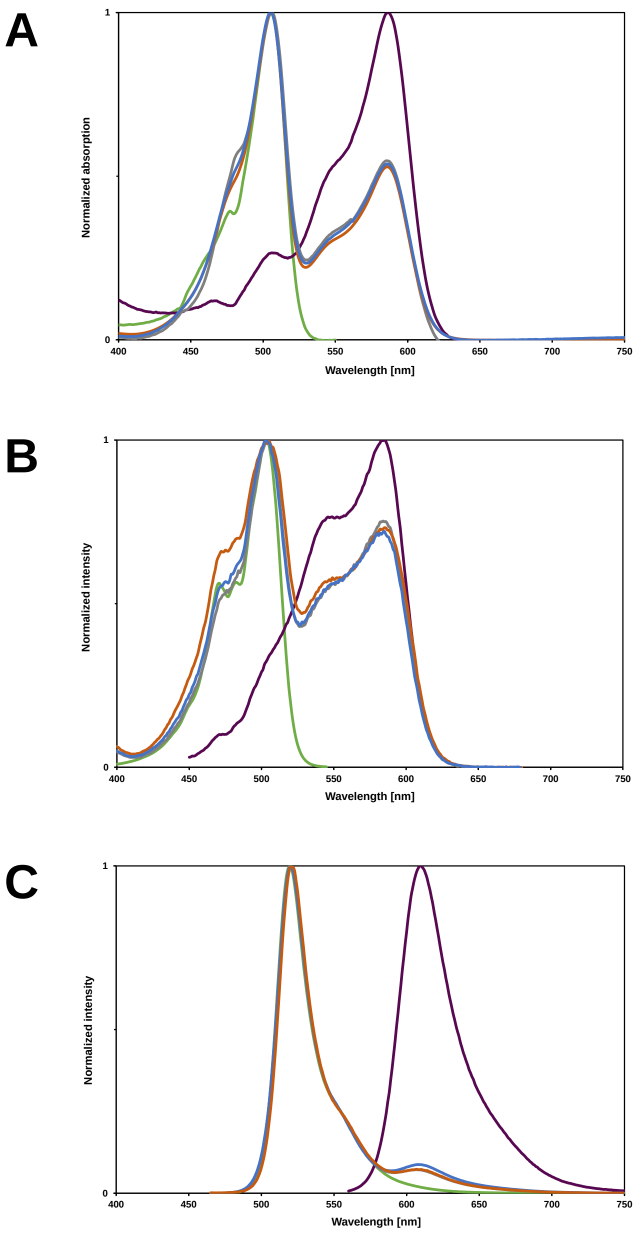

Supplementary Figure 1. Normalized absorption (A), excitation (B), and emission spectra (C) of studied fluorescent proteins. Spectra of the isolated proteins mNeonGreen (depicted by green line), mCherry (purple), mCherry-mNeonGreen (dark gray), mCherry-M1-mNeonGreen (orange), and mCherry-M10Q-mNeonGreen (blue) were normalized with respect to the highest absorbance or peak value measured. The excitation spectra were recorded with the emission wavelength set to 560 nm (mNeonGreen) or 700 nm (FRET tandem constructs, mCherry). The emission spectra were recorded using an excitation wavelength of 450 nm (mNeonGreen, FRET tandem constructs) or 545 nm (mCherry). All measurements were performed in ultra-micro quartz cuvettes with a path length of 0.3 cm at room temperature.

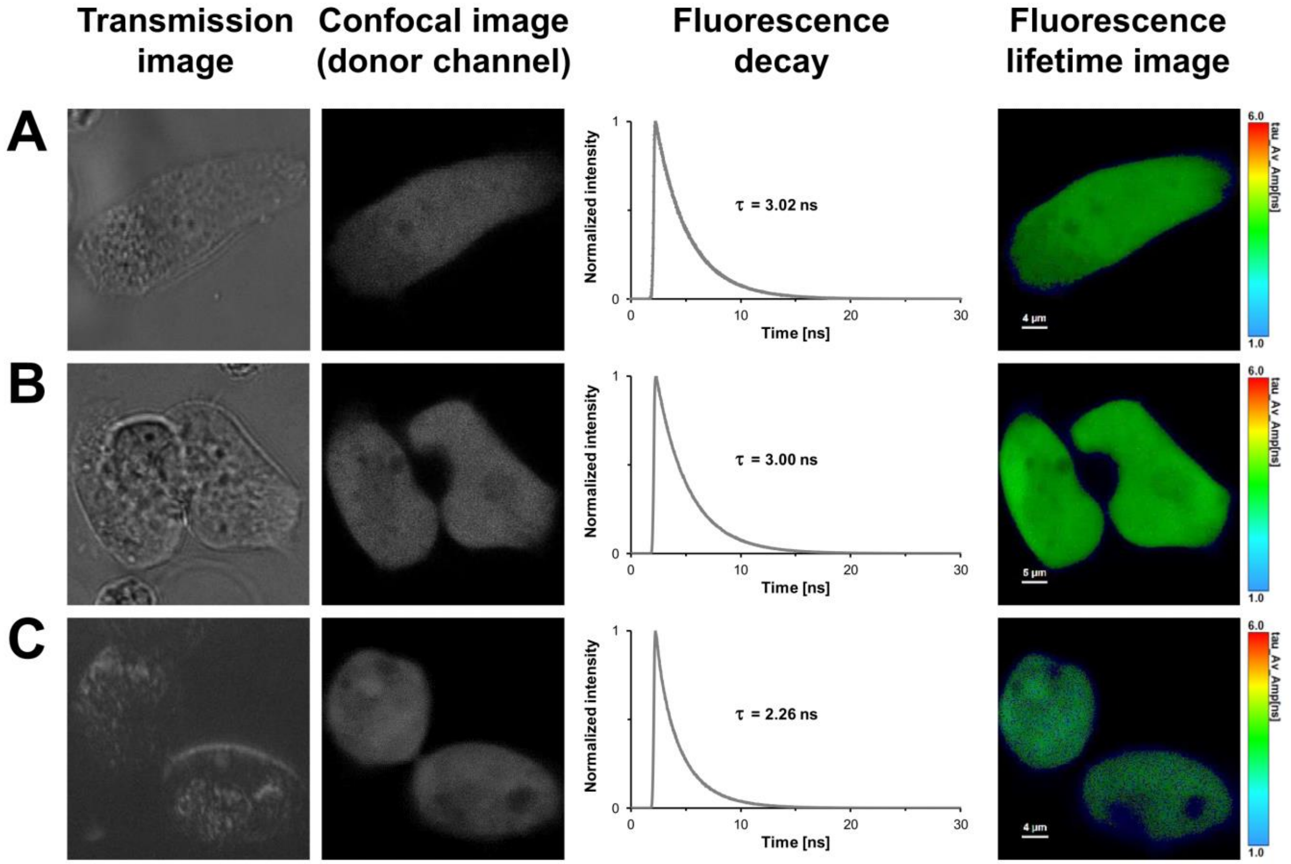

Supplementary Figure 2. FLIM-FRET-based control measurements of Hek293 cells expressing the donor mNeonGreen in absence or presence of the acceptor mCherry, and the positive FRET control. In the first column, representative transmission images of Hek293 cells expressing mNeonGreen (A), unfused mNeonGreen and unfused mCherry (B), and the positive FRET control mNeonGreen-mCherry (C) are shown. In the second column, grayscale confocal images are displayed that were obtained in the donor channel by exciting at 488 nm and recording fluorescence within a detection band of 500 - 550 nm. The third column shows the fluorescence decay curves of the cells. In the last column, fluorescence lifetime images are shown, in which lifetimes are encoded by color as specified by the color scale. Lengths of scale bars are indicated.

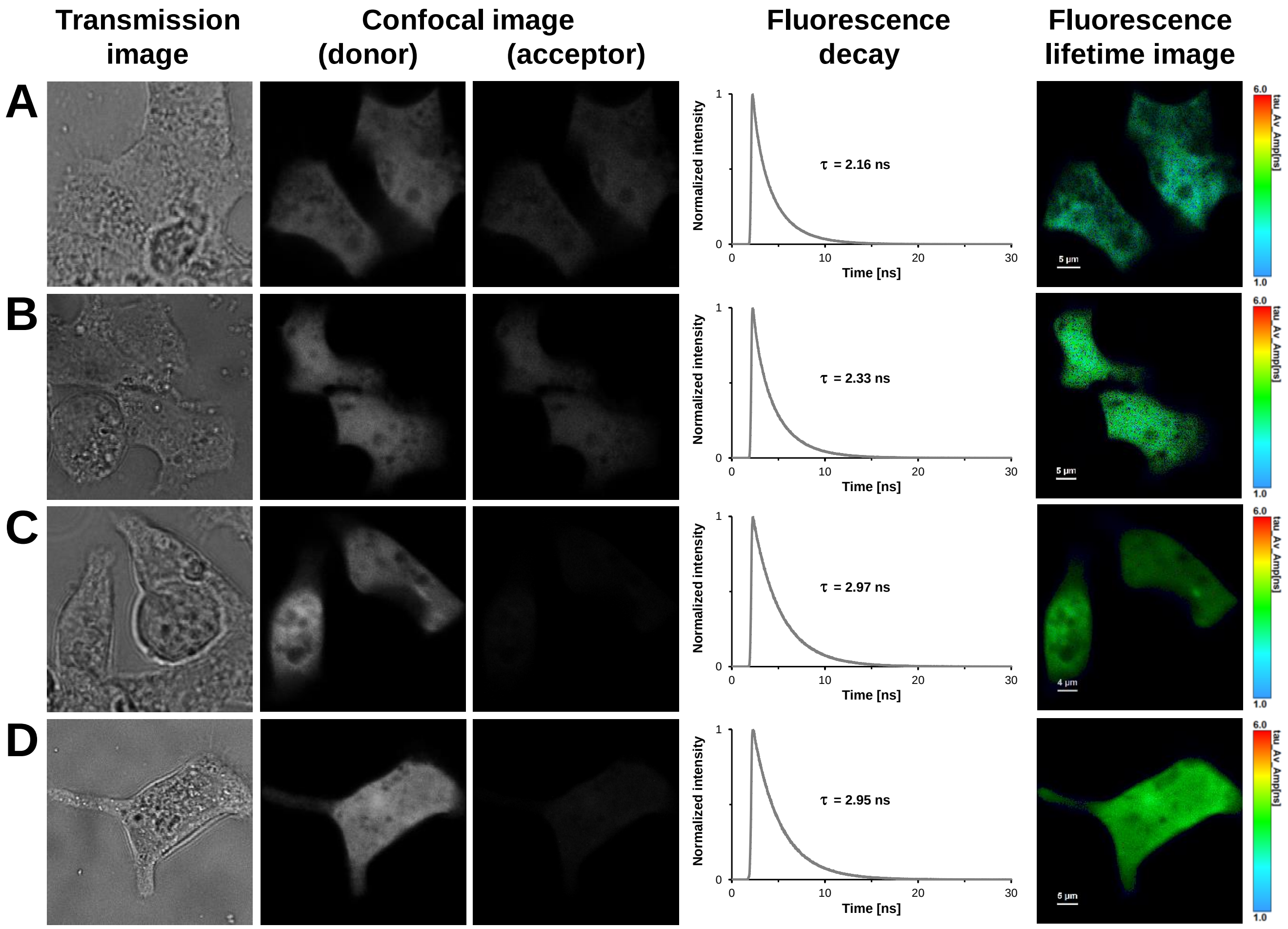

Supplementary Figure 3. FLIM-FRET-based measurements of Hek293 cells expressing FRET tandem constructs with different translation initiation sites. In the first column, representative transmission images of Hek293 cells expressing mCherry-M1-mNeonGreen (A), mCherry-M10-mNeonGreen (B), mCherry-M17-mNeonGreen (C), and mCherry-M23-mNeonGreen (D) are shown. The second and third columns show grayscale confocal images that were obtained in the donor and acceptor channel by recording fluorescence intensities within a detection band of 500 - 550 nm and 580 - 630 nm, respectively. The fourth column shows the fluorescence decay curves of the cells. In the last column, fluorescence lifetime images are shown, in which lifetimes are encoded by color as specified by the color scale. Lengths of scale bars are indicated.
